# mHealth Interventions for Health System Strengthening in China: A Systematic Review

**DOI:** 10.2196/mhealth.6889

**Published:** 2017-03-16

**Authors:** Maoyi Tian, Jing Zhang, Rong Luo, Shi Chen, Djordje Petrovic, Julie Redfern, Dong Roman Xu, Anushka Patel

**Affiliations:** ^1^ The George Institute for Global Health at Peking University Health Science Center Beijing China; ^2^ Sydney Medical School The George Institute for Global Health, Australia University of Sydney Sydney Australia; ^3^ School of Public Health Peking University Health Science Center Beijing China; ^4^ Medical School University of Michigan Ann Arbor, MI United States; ^5^ Sun Yat-sen Global Health Institute School of Public Health Sun Yat-sen University Guangzhou China

**Keywords:** mHealth, China, health care systems

## Abstract

**Background:**

With rapidly expanding infrastructure in China, mobile technology has been deemed to have the potential to revolutionize health care delivery. There is particular promise for mobile health (mHealth) to positively influence health system reform and confront the new challenges of chronic diseases.

**Objective:**

The aim of this study was to systematically review existing mHealth initiatives in China, characterize them, and examine the extent to which mHealth contributes toward the health system strengthening in China. Furthermore, we also aimed to identify gaps in mHealth development and evaluation.

**Methods:**

We systematically reviewed the literature from English and Chinese electronic database and trial registries, including PubMed, EMBASE, Cochrane, China National Knowledge of Infrastructure (CNKI), and World Health Organization (WHO) International Clinical Trials Registry Platform. We used the English keywords of mHealth, eHealth, telemedicine, telehealth, mobile phone, cell phone, text messaging, and China, as well as their corresponding Chinese keywords. All articles using mobile technology for health care management were included in the study.

**Results:**

A total of 1704 articles were found using the search terms, and eventually 72 were included. Overall, few high quality interventions were identified. Most interventions were found to be insufficient in scope, and their evaluation was of inadequate rigor to generate scalable solutions and provide reliable evidence of effectiveness. Most interventions focused on text messaging for consumer education and behavior change. There were a limited number of interventions that addressed health information management, health workforce issues, use of medicines and technologies, or leadership and governance from a health system perspective.

**Conclusions:**

We provide four recommendations for future mHealth interventions in China that include the need for the development, evaluation and trials examining integrated mHealth interventions to guide the development of future mHealth interventions, target disadvantaged populations with mHealth interventions, and generate appropriate evidence for scalable and sustainable models of care.

## Introduction

### Burden of Disease and Health System in China

In the last decade, China has undergone a continuing epidemiological transformation from infectious diseases to chronic and noncommunicable diseases (NCDs) [1,2]. NCDs caused over 80% of China’s total disability-adjusted life years in 2013 and accounted for China’s largest burden of disease [3]. Chronic and NCDs pose special challenges to existing health systems as the long-term ongoing management of such conditions requires a shift from institutional care to community-based care, with an increased focus on self-management with or without peer or family support [4]. Despite the four major rounds of health care reforms since mid-1980s in China, many health equity and system level challenges remain [4,5]. Responding to those challenges, the health system needs to be adjusted to provide more effective solutions. The portability and connectivity of mobile health (mHealth) can potentially serve as an effective tool in facilitating this adjustment and to allow the health care delivery to reach hard-to-reach population. mHealth has been variably defined. The World Health Organization (WHO) definition is medical and public health practice supported by mobile devices, such as mobile phones, personal digital assistants (PDAs), and other wireless devices [6]. mHealth involves the use of a wide range of functionalities incorporated by such mobile devices, including standard voice, short message service (SMS), Web browsing, and applications on different operating systems.

### Chinese Mobile Market and the Potential for mHealth

The unprecedented uptake of mobile phones with an ever growing telecommunications infrastructure has driven the development of mHealth innovation around the globe. In China, mobile phone penetration reached 94.5 per 100 people in 2014 [7]. Cellular signals now cover almost all residential areas from densely populated cities to remote villages, with increasing penetration of 3G and 4G networks. Penetration of smartphones has also increased rapidly, reaching 90% in urban areas and 32% in rural areas in 2015 [8]. The rapid development of this mobile infrastructure has created significant potential for mHealth interventions in China.

The rapid adoption of mobile phones may be explained by the diffusion of innovation theory, which is one of the most popular theories for studying adoption of information technologies and understanding how information technology innovations spread within and between communities [9].

### Prior Work and Objectives

Although there were several reviews documenting the mHealth interventions in low- and middle-income countries (LMICs) [10-12], no systematic reviews of the scope and value of mHealth initiatives in the largest developing country exist. The specific aims of this systematic review were to (1) characterize mHealth interventions across all disease areas in China, (2) evaluate the extent to which mHealth interventions focus on health system strengthening, and (3) identify gaps in mHealth intervention development and evaluation that need to be addressed in the future.

## Methods

### Database Search

A systematic search of the literature in both Chinese and English published from May 26, 2008 to December 17, 2015, was performed following Preferred Reporting Items for Systematic Reviews and Meta-Analyses (PRISMA) guidelines [13] using the following electronic databases: PubMed, EMBASE, Cochrane, and China National Knowledge of Infrastructure (CNKI). We also searched for registered trials in the WHO International Clinical Trials Registry Platform, which included 15 approved trial registries and supplementary searches in Chinese Clinical Trial Registry (CHICTR), and Clinicaltrials.gov. English keywords used in these searches included the following: mHealth, eHealth, telemedicine, telehealth, mobile phone, cell phone, text messaging, and China. The Chinese keywords used include “ShouJi” (mobile phone or cell phone), “DuanXin” (text messaging), “YiDongJiangKang” (mHealth), and “Yi Dong Yi Liao” (mobile medicine). Multimedia Appendix 1 lists the detailed search strategy for each database.

### Inclusion and Exclusion Criteria

We included all articles related to health care management using mobile technology in China. Any type of the following articles with full texts was included: (1) randomized controlled trials (RCTs), (2) quasi-experimental studies, (3) descriptive studies without any outcome measured, or (4) registered RCTs. We only included studies written in English or Chinese, and articles related to telemedicine or telehealth were only included if mobile technologies were used as part of the intervention. We excluded all articles describing technology development, review articles, protocol papers, and any studies using fixed landline phone or the Internet using a desktop computer as part of the intervention. A total of 5 reviewers independently evaluated and excluded articles at the abstract review stage. Full-text articles whose abstracts met the inclusion criteria were then reviewed by 3 reviewers.

### Analytical Framework

**Figure 1:**
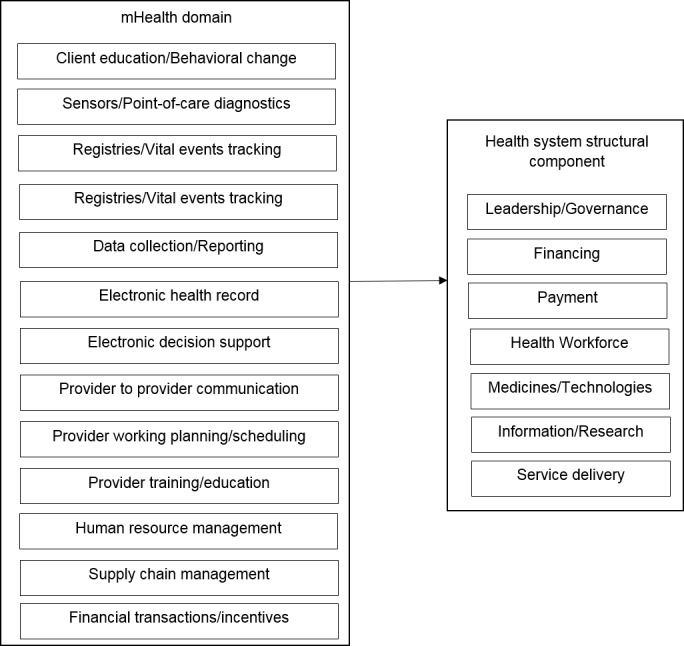
Adapted health system framework for evaluating mHealth interventions.

We utilized an adapted health system framework to evaluate the role of mHealth interventions as a health system strengthening tool (Figure 1) [14-16]. In this framework, there were two dimensions: (1) the function of mHealth intervention categorizing into one of the 12 mHealth tools proposed by Labrique et al [14], and (2) the corresponded health system frame work as developed by Hsiao and WHO [15,16]. Assessing both dimensions of the mHealth intervention allowed us to identify where the gaps were in the mHealth interventions from a health systems perspective.

### Data Extraction

A spreadsheet was developed for entering extracted data that included study characteristics, the mHealth domain, and the health system domain using the aforementioned analytical framework [16]. An agreement was reached on the definitions and interpretation of each variable in the data extraction template among the reviewers before data collection. Three reviewers independently extracted the data into the template and cross-reviewed. Disagreements in this step were resolved by consensus.

### Quality Assessment

For RCTs, methodological quality was assessed using the Cochrane Risk of Bias Assessment Tool [17]. We assessed the random sequence generation, allocation concealment, blinding of participants, personnel and outcome assessors, incomplete outcome data, selective outcome reporting, and other sources of bias. Any discrepancies in article inclusion, data extraction, and bias assessment were discussed and resolved by team consensus.

## Results

### Included Studies

**Figure 2:**
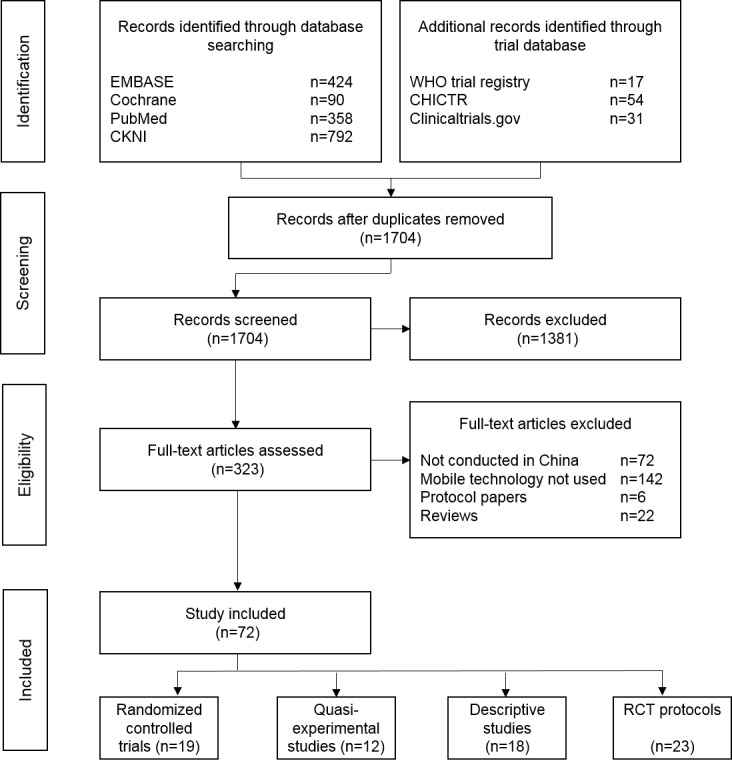
Study flowchart.

We retrieved 1704 articles using the search terms, and 323 articles were selected for full-text review (Figure 2). Of those, 251 studies were excluded for the following reasons: not conducted in China (n=81), not using the mobile technology (n=142), protocol papers (n=6), and review articles (n=22).

### Study Characteristics

The study characteristics, mHealth domain, and health system domain of the nonprotocol articles (n=49) are summarized in Table 1. The majority of the studies were conducted in an urban setting (n=34) [18-51], with only 6 focusing on a rural population [28,51-55]. The most common disease focus was on NCDs (n=15) [22,25,26,29,30,34,37-39,42,45,46,52,56,57], whereas 12 studies focused on infectious diseases [33,41,51,53,54,58-64] and 8 studies were designed for maternal and child health [36,40,43,47-49,55,65]. A wide range of study designs was used to evaluate or describe the mHealth intervention, including 18 exploratory studies that described, validated, or pilot-tested mHealth interventions without any quantitative outcome assessment [18-28,58-62,64,66]. A total of 31 studies quantitatively evaluated the mHealth intervention [29-57,63,65], of which 19 utilized a RCT design [29-35,38-40,43,47,51-53,56,57,63,65] whereas the remainder used a quasi-experimental study design (n=12). In most cases, the primary mobile technology was a regular mobile phone (n=36) [18,19,21,25,29-49,51,53-57,59,62-65]. Only 12 studies utilized smartphone technology for the intervention [20,22-24,26-28,50,52,58,61,66].

**Table 1:** Study characteristics, mHealth domain, and health system domain of nonprotocol articles.

Author	Setting	Disease area	Population (n)	Study description		Type of device	mHealth domain	Health system domain
**Descriptive Studies**									
Deng [18]	Urban	Others -patients for sedation gastrointestinal endoscopy (SGIE)	908 outpatients in the anesthesia clinic for SGIE	Feasibility to use SMS to improve the adherence for SGIE appointment		R^	Client education and behavior change	Service delivery
Chen [19]	Urban	Others -suicide attempters	15 suicide attempters from the emergency department	Feasibility to SMS to decrease recidivism for suicide attempters		R	Client education and behavior change	Service delivery
Li [58]	Not described	Infectious disease	Not described	A decision support system for the responses to infectious disease emergencies		S*	Electronic decision support	Leadership/governance
Zhao [20]	Urban	Not mentioned	Not described	A case report describing development of a shared community health information system		S	Electronic medical record	Leadership/governance
Li [59]	Not described	Infectious disease -hand, foot, and mouth disease	Not described	Use of SMS to develop automated alert and response system for hand, foot, and mouth disease		R	Registries and vital event tracking	Leadership/governance
Guo [60]	Not described	Infectious disease	Not described	A mobile phone-based infectious disease reporting system in earthquake-affected area		PDA^a^	Data collection and reporting	Information
Mao [21]	Urban	Not mentioned	100 patients admitted from general hospital	Use of SMS to deliver individualized pharmaceutical care		R	Client education and behavior change	Service delivery
Yang [61]	Not described	Infectious disease	495 health care agencies in earthquake-affected area	Use of mobile phone as a surveillance tool to monitor infectious disease		S	Data collection and reporting	Information
Jun [22]	Urban	Noncommunicable disease -adolescent Idiopathic Scoliosis	64 adolescent idiopathic scoliosis patients	Use of smartphone to measure the axial trunk rotation		S	Sensors and point-of-care diagnosis	Medicines/technologies
Zhang [64]	Not described	Infectious disease -schistoscomajaponicum infection	Not described	Use of SMS to send alert the fishermen to avoid the schistosome infection		R	Registries and vital event tracking	Leadership/governance
Ma [62]	Not described	Infectious disease	Not described	Development of SMS-based emergency response system for infectious disease		R	Registries and vital event tracking	Leadership/governance
Guan [23]	Urban	Others -voiding diary monitoring	20 healthy volunteers	Development of smartphone-based remote voiding diary monitoring system		S	Data collection and reporting	Service delivery
Ye [24]	Urban	Others -slitlampbiomicroscopy	Not described	Use of smartphone camera for teleophthalmology		S	Sensors and point-of-care diagnosis	Service delivery
Yu [66]	Not described	Not mentioned	11 volunteers	Health examination toolkit involving sensors and data upload into an Android phone		S	Sensors and point-of-care diagnosis	Service delivery
Yin [25]	Urban	Noncommunicable disease -dialysis patients	Not described	Development of mobile phone-based follow up system		R	Client education and behavior change	Service delivery
Yang [65]	Urban	Noncommunicable disease -facial acne	80 patients with facial acne	Use of mobile phone to grade the severity of facial acne		S	Sensors and point-of-care diagnosis	Service delivery
Wang [27]	Urban	Others -dietary intake assessment	35 healthy volunteers	Development of dietary intake assessment using mobile phone camera function		S	Data collection and reporting	Medicines/technologies
Smith [28]	Rural and urban	Not mentioned	110 healthy adults	Development of a smartphone-assisted 24-h recall to assess beverage consumption		S	Data collection and reporting	Medicines/technologies
**RCT**				**Intervention**	**Follow-up**			
Tian [52]	Rural	Noncommunicable disease -cardiovascular disease	2086 high cardiovascular risk patients	A smartphone based electronic decision support system focusing on two medication use and two lifestyle modifications	12 month	S	Electronic decision support	Service delivery
Lin [29]	Urban	Noncommunicable disease -obesity	123 overweight adults	SMS-assisted lifestyle weight loss intervention	6 month	R	Client education and behavior change	Service delivery
Liu [51]	Rural and urban	Infectious disease -tuberculosis	4173 pulmonary TB^b^ patients	SMS reminders and medication monitoring	6 month	R	Client education and behavior change	Service delivery
Sabin [63]	Not described	Infectious disease -HIV^c^	120 HIV patients	Real time SMS reminders triggered by the electronic medication storage device	6 month	R	Client education and behavior change	Service delivery
Liu [30]	Urban	Noncommunicable disease -cardiovascular disease	589 workers without known CVD^d^	Mobile-phone based lifestyle intervention	12 month	R	Client education and behavior change	Service delivery
Shi [31]	Urban	Others -smokers	179 adolescent smokers	Smoking cessation lifestyle intervention delivered by the SMS	12 week	R	Client education and behavior change	Service delivery
Chen [53]	Rural	Infectious disease -Viral infections affecting upper respiratory tract and otitis media	977 township level health workers	SMS based health worker training	1 month	R	Provider training and education	Health workforce
Deng [32]	Urban	Others -outpatients for sedation gastrointestinal endoscopy	2200 outpatients	SMS reminders to attend medical examination	Not mentioned	R	Client education and behavior change	Service delivery
Lv [56]	Not described	Noncommunicable disease -asthma	150 outpatients with asthma	SMS reminders for asthma self-management	12 week	R	Client education and behavior change	Service delivery
Wang [57]	Not described	Noncommunicable disease -allergic rhinitis	50 outpatients with allergic rhinitis	SMS reminders to improve adherence to medication and treatment	30 days	R	Client education and behavior change	Service delivery
Chai [33]	Urban	Infectious disease -H1N1	1992 residents in Shanghai	SMS-based health education for H1N1 prevention	10 days	R	Client education and behavior change	Service delivery
Lin [65]	Not described	Maternal and child health	258 parent-child pairs with child having cataract	SMS reminders to attend medical appointment	4 days	R	Client education and behavior change	Service delivery
Dai [34]	Urban	Noncommunicable disease -diabetes	80 type-2 diabetes patients	SMS based health education	12 month	R	Client education and behavior change	Service delivery
Shi [35]	Urban	Others -smokers	176 adolescent smokers	SMS based health education for smoking cessation	3 month	R	Client education and behavior change	Service delivery
Zhang [40]	Urban	Maternal and child health	166 children with asthma	SMS-based health promotion	3 month	R	Client education and behavior change	Service delivery
Wei [38]	Urban	Noncommunicable disease -chronic kidney disease	108 patients with chronic kidney disease	SMS-based medication adherence intervention	3 month	R	Client education and behavior change	Service delivery
Li [43]	Urban	Maternal and child health	82 pregnant women	SMS-based dietary recommendation during pregnancy	Not mentioned	R	Client education and behavior change	Service delivery
Chen [74]	Urban	Maternal and child health	155 pregnant women	SMS-based breastfeeding promotion	16 week	R	Client education and behavior change	Service delivery
Qu [25]	Urban	Noncommunicable disease -schizophrenia	178 patients with schizophrenia	SMS-based medication adherence intervention	12 month	R	Client education and behavior change	Service delivery
**Quasi-experiment**									
Jiang [49]	Urban	Maternal and child health	582 expectant mothers	SMS-based intervention about infant feeding	12 month	R	Client education and behavior change	Service delivery
Fang [42]	Urban	Noncommunicable disease -hypertension	599 hypertensive patients	SMS-based health education for hypertension management	12 month	R	Client education and behavior change	Service delivery
Zhao [46]	Urban	Noncommunicable disease -diabetes	64 type-2 diabetes patients	SMS-based medication adherence and health education program	3 month	R	Client education and behavior change	Service delivery
Qin [44]	Urban	Others -dialysis	92 dialysis patients	SMS-based health education for dialysis patients delivered by the nurse	53-612 days	R	Client education and behavior change	Service delivery
Xie [45]	Urban	Noncommunicable disease -diabetes	196 type-2 diabetes patients	SMS-based health promotion for diabetes management	12 month	R	Client education and behavior change	Service delivery
Chen [54]	Rural	Infectious disease -schistosomiasis	501 healthy residents	SMS-based health promotion for schistosomiasis prevention	10 month	R	Client education and behavior change	Service delivery
Chen [48]	Urban	Maternal and child health	180 children with allergic rhinitis	SMS-based health education for allergic rhinitis management	12 month	R	Client education and behavior change	Service delivery
Xu [41]	Urban	Infectious disease -HIV	71 HIV patients	SMS-based medication adherence intervention	12 month	R	Client education and behavior change	Service delivery
Ni [36]	Urban	Maternal and child health	460 pregnant women	SMS-based health education	5 month	R	Client education and behavior change	Service delivery
Liu [37]	Urban	Noncommunicable disease -acute coronary syndrome	82 ACS^e^ patients	SMS based medication adherence intervention	1 month	R	Client education and behavior change	Service delivery
Zhou [55]	Rural	Maternal and child health	N250 pregnant women	SMS-based health education for HIV prevention	1 month	R	Client education and behavior change	Service delivery
He [50]	Urban	Others -general health	100 residents with smartphone	Smartphone-based pedometer “app”	6 months	S	Sensors and point-of-care diagnosis	Service delivery

^a^PDA: personal digital assistant.

^b^TB: tuberculosis.

^c^HIV: human immunodeficiency virus.

^d^CVD: cardiovascular disease.

^e^ACS: acute coronary syndrome.

^^^R: regular mobile phone.

^*^S: smartphone.

The search of registered clinical trials identified 23 additional mHealth registered RCTs (Multimedia Appendix 2). Although 12 of these studies were listed as completed, we were only able to find 5 studies with published results. All 5 studies were identified during the original systematic review of the literature [29,32,51,52,65]. Consistent with the published RCTs, the majority of the interventions described in the registry focused on client education and behavior change using simple text messaging.

### Role of mHealth in the Health System

Applying the adapted health system framework (Table 2), we found the client education and behavioral change communication was the most commonly targeted mHealth domain (n=32) [18,19,21,25,29-49,51,54-57,63,65]. It was found that 5 interventions addressed sensors and point-of-care diagnostics [22,24,26,50,66], 5 interventions focused on data collection and reporting [23,27,28,60,61], 3 interventions involved registries and vital events tracking [59,62,64], 2 interventions focused on electronic decision support [52,58], 1 intervention involved electronic health records [20], and 1 intervention delivered provider training and education [53]. There were no interventions identified in the domains of provider to provider training, provider work planning and scheduling, human resources management, supply chain management, or financial transactions and incentives. From a health systems perspective, most studies targeted service delivery (n=38) [18,19,21,23-26,29-52,54-57,63,65,66]. Few interventions focused on the provision or management of information (n=2) [60,61], health workforce support (n=1) [53], medicines and technologies (n=3) [22,27,28], or leadership and governance (n=5) [20,58,59,62,64].

### Risk of Bias Assessment

For the RCTs, risk of bias was mostly classified as either low or unclear (Table 3). Four studies did not provide sufficient information to assess risk [34,35,43,47].

**Table 2:** Health system framework assessment of the mHealth interventions.

mHealth Functionality	Health System Structural Component							
	Leadership/ Governance	Financing	Payment	Health Workforce	Medicines/ Technologies	Information	Service Delivery	Sub-total
Education/behavioral							32	32
Sensors/point-of-care devices					1		4	5
Registries/vital events tracking	3							3
Data collection and reporting					2	2	1	5
Electronic health records	1							1
Electronic decision support	1						1	2
Provider to provider communication								
Provider work planning/scheduling								
Provider training/education				1				1
Human resources management								
Supply chain management								
Financial transactions/incentives								
Sub-total	5			1	3	2	38	

**Table 3:** Risk of bias assessment for randomized controlled trials.

Author	Sequence generation	Allocation concealment	Blinding of participants, personnel, and outcome assessors	Incomplete outcome data	Selective outcome reporting	Other sources of bias
Tian [52]	Low	Low	Low	Low	Low	Low
Lin [29]	Low	Low	Low	Low	Unclear	Low
Liu [51]	Low	Unclear	Unclear	Unclear	Unclear	Low
Sabin [63]	Low	Low	Unclear	Low	Unclear	Low
Liu [30]	Low	Low	Low	Low	Unclear	Low
Shi [31]	Unclear	Unclear	Unclear	Low	Unclear	Low
Chen [53]	Low	Low	Low	Low	Unclear	Low
Deng [32]	Low	Low	Low	Unclear	Unclear	Low
Lv [56]	Low	Unclear	Unclear	Unclear	Unclear	Low
Wang [57]	Low	Low	Low	Unclear	Unclear	Low
Chai [33]	Low	Unclear	Low	Unclear	Unclear	Low
Lin [65]	Low	Low	Low	Low	Unclear	Low
Dai [34]	Unclear	Unclear	Unclear	Unclear	Unclear	High
Shi [35]	Unclear	Unclear	Unclear	Unclear	Unclear	High
Zhang [40]	Low	Unclear	Unclear	Unclear	Unclear	Unclear
Wei [38]	Low	Unclear	Unclear	Unclear	Unclear	Unclear
Li [43]	Unclear	Unclear	Unclear	Unclear	Unclear	High
Chen [47]	Unclear	Unclear	Unclear	Unclear	Unclear	High
Qu [39]	Low	Low	Low	Low	Unclear	Low

## Discussion

### Principal Findings

In this study, we reviewed studies and registered trials for studies published in the peer-reviewed journals involving mHealth interventions in China. We particularly focused on the extent to which mHealth interventions had the capacity to contribute to health care strengthening in the context of a rapidly evolving disease burden. Although we did observe an increasing focus on NCDs, there was little evidence of the development of mHealth interventions that were likely to substantially strengthen health care systems. We also noted a large disparity in the development of mHealth interventions that were focused on rural as opposed to urban areas. In addition, the quality of evidence provided in relation to effectiveness of such interventions is generally poor.

### Comparison With Other Reviews

Beratarrechea et al [11] conducted a review to examine the role of mHealth intervention on the management of NCDs in LMICs, with a focus on the use of SMS and automated voice interventions. The study found that there were significant improvement on certain clinical outcomes and processes of care. Peiris et al further performed a review to explore the impact of all mHealth interventions on health care quality for NCDs in LMICs. Similar to our findings, there were few high-quality studies, and most of the studies used the SMS for patient behavior change. Very few studies addressed the mHealth intervention as a health system strengthening tool.

### Health System Strengthening

On the basis of the literature we have identified, the development of mHealth interventions by academia in China remains relatively under-developed, in terms of both scope and capability. Interventions mostly utilized a texting tool to provide client education and behavior change. We identified a focus on only 7 of the 12 mHealth domains, with no interventions concentrating on interprovider communication or health service management, including financial transactions. In addition, all the interventions were developed as stand-alone tools to deliver health services, with little or no exploration of how integration within existing or developing health systems can be achieved.

### Health Equality

Equitable access to quality health services is an important dimension of an effective health system. In China, around 50% of the population is based in rural regions, where health outcomes are, in general, poorer than those among urban communities. Addressing such inequities is a public health priority, and mHealth strategies may provide a particular opportunity to reduce gaps that relate to weaker health systems. As China’s mobile network reaches far and deep into its rural areas, mHealth solutions provide a real opportunity to strengthen rural health systems. Despite the huge potentials of mHealth help in closing the health equity gap, few academic studies in China has chosen to focus on this area. The regional imbalance identified in this review may be explained by the greater convenience of conducting studies in urban communities. However, the potential for mHealth to impact on health outcome inequities cannot be addressed if the digital gulf between those who have access to mobile technology in urban areas and those who do not have access in rural areas is not reduced. Similar considerations are relevant to other disadvantaged subgroups of population, including those with relatively low literacy or socioeconomic status.

### Quality of Evidence

A key objective of mHealth research should be to provide useful and reliable evidence for end users, including policy-makers in the context of those innovations aimed at improving health outcomes through deployment in the public health care system. Our review found that published and planned mHealth studies in China largely have not and will not produce such outcomes. Fewer than 40% of the published studies utilized an RCT design and all were of uncertain or poor quality based on objective measures. The majority of the reports were descriptive, with no apparent attempt to determine efficacy or effectiveness. Study outcomes were largely the product of low-quality and small-scale experiments, which provided little understanding of the true impact of an intervention with large-scale real-world implementation within complex health systems.

### Limitations

There are several limitations to this review. Firstly, we were not able to conduct a quantitative meta-analysis of the outcomes due to the heterogeneity of the RCTs. We identified a number of ongoing trials from the trial registry. The published results of those trials will enable to provide increased power to determine the size of the effect of mHealth interventions on health outcomes. Second, although the adapted health system framework was useful to evaluate the mHealth intervention as a health system strengthening tool, a single study may address multiple mHealth domains or health system domains. We only reported the primary functionality of the mHealth intervention and the key aspect that the intervention addressed in the health system. Finally, this review mainly targeted academic studies in the literature. We should note that China is experiencing rapid development in mHealth technology in the commercial world, many of which may have health system implications that we had limited ability to evaluate in this review.

### Conclusions

mHealth has the potential to overcome some of the challenges due to the rapid changing environment of health care needs and provision in China. However, this potential can only be realized through the continual development of mHealth interventions to strengthen the health system, utilizing a subsequent rigorous approach to generating high-quality evidence about the likely implications of “real world implementation.” Therefore, we outline three recommendations for future mHealth research and development in China: (1) mHealth studies should not be conducted as the standalone technical study evaluating its efficacy in the vacuum of the social context, (2) promote the development of integrated mHealth interventions as a tool to serve the existing health system, (3) focus on developing and evaluating mHealth interventions with the potential to reduce health outcome disparities within the population, and (4) conduct large-scale rigorously designed “real world” evaluation of mHealth interventions focused on health system strengthening. Specific public and private investment into such research is a priority.

## Appendix

Multimedia Appendix 1 Detailed search strategy for each database used.

Multimedia Appendix 2 Table: Registered randomized controlled trials in clinical trials database.

## Abbreviations

ACS: acute coronary syndrome
CHICTR: Chinese Clinical Trial Registry
CNKI: China National Knowledge of Infrastructure
COPD: chronic obstructive pulmonary disease
CVD: cardiovascular disease
HIV: human immunodeficiency virus
LMICs: low- and middle-income countries
NCDs: noncommunicable diseases
PDA: personal digital assistant
PRISMA: Preferred Reporting Items for Systematic Reviews and Meta-Analyses
RCTs: randomized controlled trials
SGIE: sedation gastrointestinal endoscopy
SMS: short message service
TB: tuberculosis
WHO: World Health Organization
