# Supplementary material for: mHealth Interventions for Health System Strengthening in China: A Systematic Review
Source: JMIR Mhealth Uhealth. 2017 Mar 16;5(3):e32. doi: 10.2196/mhealth.6889 (PMC5374274; doi:10.2196/mhealth.6889)
Supplement: Multimedia Appendix 1 [file mhealth_v5i3e32_app1.pdf]

Appendix 1: Detailed search strategy for each database used

**DATABASE/REGISTRY**	**RESEARCH STRATEGY**
Pubmed	(ehealth[MeSH Terms] OR mhealth[MeSH Terms] OR telemedicine[MeSH Terms] OR text messaging[MeSH Terms] OR mobile phone[MeSH Terms]) AND China
Embase	chin*:ab,ti AND ('telehealth'/exp OR 'mhealth' OR 'ehealth' OR 'telemedicine' OR 'telehealth' OR 'mobile phone'/exp OR 'mobile phone' OR 'text messaging'/exp OR 'text messaging'
	'china'/exp OR 'china' AND ('telehealth'/exp OR 'telehealth' OR 'mhealth' OR 'ehealth' OR 'telemedicine' OR 'mobile phone'/exp OR 'mobile phone' OR 'text messaging'/exp OR 'text messaging') AND [review]/lim AND ([conference review]/lim OR [review]/lim OR [systematic review]/lim)
Cochrane Library	(telemedicine [MeSH Terms] OR Cell phones[MeSH Terms] OR Text messaging [MeSH Terms] OR mhealth OR ehealth OR telemedicine OR telehealth) AND chin*
Clinicaltrails.gov registry	Searched for individual terms (telemedicine, telehealth, text messaging, text message, ehealth, mhealth, mobile phone) individually with country of origin - China.
ISRCTN registry	Searched for individual terms (telemedicine, telehealth, text messaging, text message, ehealth, mhealth, mobile phone) individually with country of origin - China.
CHICTR registry	Searched for individual terms (message, text, mobile, telemedicine, telehealth, mhealth, ehealth) through all text
CNKI	(‘Shou Ji' + 'Duan Xin'+ 'Yi Dong Yi Liao' + 'Yi Dong Jian Kang') AND HX=Y NOT CLC = '+‘
