# Supplementary material for: mHealth Interventions for Health System Strengthening in China: A Systematic Review
Source: JMIR Mhealth Uhealth. 2017 Mar 16;5(3):e32. doi: 10.2196/mhealth.6889 (PMC5374274; doi:10.2196/mhealth.6889)
Supplement: Multimedia Appendix 2 [file mhealth_v5i3e32_app2.pdf]

Table 2. Registered randomized controlled trials in clinical trials database.

Database	Registration Number	Trial status	Disease area	Intervention	mHealth domain	Health system domain
ISRCTN	44149146	C[*]	Maternal and child health	Daily text messaging reminder to caregivers to feed baby the nutritional supplement	Client education and behavior change	Service delivery
Clinicaltrials.gov	NCT02189616	O[^]	NCD[a]<br>-asthma	Weekly text messages for asthma self-management	Client education and behavior change	Service delivery
Clinicaltrials.gov	NCT02626416	C	Maternal and child health	A mobile "app" used by the parents for timely detection of posterior capsular opacification	Sensors and point-of-care diagnosis	Medicines/technologies
Clinicaltrials.gov	NCT01491906	C	NCD<br>-obesity	Text messaging assisted lifestyle weight loss intervention	Client education and behavior change	Service delivery
Clinicaltrials.gov	NCT01503814	C	NCD<br>-cardiovascular disease	A smartphone based electronic decision support system focusing on two medication use and two lifestyle modifications provided by the community health workers	Electronic decision support	Service delivery
Clinicaltrials.gov	NCT02646059	O	Others<br>-blood donation	Text messaging to recruit past blood donors to donate blood again	Client education and behavior change	Service delivery
Clinicaltrials.gov	NCT01417819	C	Maternal and child health	Text message reminders to attend medical appointment	Client education and behavior change	Service delivery
Clinicaltrials.gov	NCT02037087	C	Maternal and child health	Text message to deliver good prenatal practice and care seeking	Client education and behavior change	Service delivery
Clinicaltrials.gov	NCT02589730	O	NCD<br>-diabetes	Mobile "app" for personalized diabetes management	Electronic decision support	Service delivery
Clinicaltrials.gov	NCT02140619	U[#]	NCD<br>-stroke	Text message reminders for medication adherence	Client education and behavior change	Service delivery
Clinicaltrials.gov	NCT02041390	C	Others<br>-stent implantation	Text message reminders for appropriate stent management after implantation	Client education and behavior change	Service delivery

Clinicaltrials.gov	NCT01914107	O	NCD<br>-cancer	Real time pain management provided by the clinicians via a mobile "app"	Data collection and reporting	Service delivery
Clinicaltrials.gov	NCT02140827	C	Others<br>-colonoscopy	Knowledge for bowel preparation delivered by mobile phone based social media "app"	Client education and behavior change	Service delivery
Clinicaltrials.gov	NCT02432469	O	NCD<br>-coronary artery disease	Mobile "app" for patient self-management	Client education and behavior change	Service delivery
ChiCTR	15006724	O	Infectious disease<br>-HIV[b]	Knowledge for HIV prevention through a mobile "app"	Client education and behavior change	Service delivery
ChiCTR	15006053	O	NCD<br>-mental disease	Text messaging to improve patient drug compliance	Client education and behavior change	Service delivery
ChiCTR	13003257	C	NCD<br>-COPD[c]	Mobile "app" for COPD management	Client education and behavior change	Service delivery
ChiCTR	12002635	C	NCD<br>-lumbar disc herniation	Text messaging for health education	Client education and behavior change	Service delivery
ChiCTR	12002224	C	Others<br>-outpatients for sedation gastrointestinal endoscopy	Text message reminders to attend medical examination	Client education and behavior change	Service delivery
ChiCTR	11001772	C	NCD<br>-diabetes	Mobile phone based self-monitoring	Client education and behavior change	Service delivery
ChiCTR	15006268	O	NCD<br>-mental disease	Mobile phone intervention	Not stated	Not stated
Clinicaltrials.gov	NCT01837121	U	NCD<br>-diabetes	Text message reminders for revisit appointment	Client education and behavior change	Service delivery
ISRCTN	46846388	C	Infectious disease<br>-TB[d]	Text messaging reminders and medication monitoring	Client education and behavior change	Service delivery

[a]NCD: noncommunicable disease.
[b]HIV: human immunodeficiency virus.
[c]COPD: chronic obstructive pulmonary disease.
[d]TB: tuberculosis.
[*]C: Completed
[^]O: Ongoing
[#]U: Unknown
